# 

**Published:** 1867-04

**Authors:** 


### CONTENTS.

### ORIGINAL CONTRIBUTIONS.

ART. XVII. Report on the History of Medicine. By S. J. Young, M.D., Ch'n Com., Paris, Ill., ---------- 193

ART. XVIII. Suspended Development in Twin Pregnancies. By M. M. Eaton, M.D., Peoria, Ill., ---------- 210

ART. XIX. The Interior of the Urethra viewed by a Magnesium Light. By E. Andrews, A.M., M.D., Professor of Surgery in Chicago Med. College, ---------- 211

### SELECTIONS.

A History of the Break-Bone Fever as it prevailed in the City of Montgomery, Alabama. By R. Fraser Michel, M.D., ---------- 212
The Medical Use of Electricity. By G. M. Beard, M.D., and A. D. Rockwell, M.D., New York, ---------- 225

### CORRESPONDENCE.

Chloride of Sodium in the Ulcerative Stages of Typhoid Fever, ---------- 233

### PROCEEDINGS OF SOCIETIES.

Alumni Association of Chicago Medical College, ---------- 235
Chicago College of Pharmacy, ---------- 24[illegible]

### BOOK NOTICES.

On the Action of Medicine in the System. By Frederick William Headland, M.D., B.A., F.L.S., ---------- 242
Orthopedics: A Systematic Treatise upon the Prevention and Correction of Deformities. By David Prince, M.D., ---------- 242
Contributions to the Pathology, Diagnosis, and Treatment of Angular Curvature of the Spine. By Benjamin Lee, M.D., ---------- 243
Watson Abridged: A Synopsis of the Lectures on the Principles and Practice of Physic, delivered at King's College, London. By Thomas Watson, M.D., ---------- 243
The Science and Practice of Medicine. By William Aitken, M.D., Edin., Prof. of Pathology in the Army Medical School, ---------- 244
Medical Register of the District of Columbia, for 1867. By J. M. Toner, M.D., ---------- 245

### EDITORIAL.

Convention of Delegates from Medical Colleges, ---------- 245
Public Commencement of Chicago Medial College, ---------- 249
Alumni Association, ---------- 250
American Medical Association, ---------- 251
Money Receipts for Examiner, from Feb. 21st to Mar. 22d, ---------- 254
Mortality Report for the Month of February, ---------- 253
Havard Medical College, ---------- 209
Milk Consumed in New York and Vicinity, ---------- 209
Death of Dr. W. Brinton, ---------- 209

